# Study on Platinum Coating Depth in Focused Ion Beam Diamond Cutting Tool Milling and Methods for Removing Platinum Layer

**DOI:** 10.3390/ma8095317

**Published:** 2015-09-22

**Authors:** Woong Kirl Choi, Seung Yub Baek

**Affiliations:** 1 School of Mechanical Engineering, Inha University, 253 Yonghyun-Dong, Nam-Gu, Incheon 402-751, Korea; E-Mail: xiongjie_123@hotmail.com; 2 Department of Mechanical Design, Induk University, Wolgye 2-Dong, Nowongu, Seoul 139-749, Korea

**Keywords:** focused ion beam (FIB), platinum coating, ultra-precision grinding, etching, aqua regia

## Abstract

In recent years, nanomachining has attracted increasing attention in advanced manufacturing science and technologies as a value-added processes to control material structures, components, devices, and nanoscale systems. To make sub-micro patterns on these products, micro/nanoscale single-crystal diamond cutting tools are essential. Popular non-contact methods for the macro/micro processing of diamond composites are pulsed laser ablation (PLA) and electric discharge machining (EDM). However, for manufacturing nanoscale diamond tools, these machining methods are not appropriate. Despite diamond’s extreme physical properties, diamond can be micro/nano machined relatively easily using a focused ion beam (FIB) technique. In the FIB milling process, the surface properties of the diamond cutting tool is affected by the amorphous damage layer caused by the FIB gallium ion collision and implantation and these influence the diamond cutting tool edge sharpness and increase the processing procedures. To protect the diamond substrate, a protection layer—platinum (Pt) coating is essential in diamond FIB milling. In this study, the depth of Pt coating layer which could decrease process-induced damage during FIB fabrication is investigated, along with methods for removing the Pt coating layer on diamond tools. The optimum Pt coating depth has been confirmed, which is very important for maintaining cutting tool edge sharpness and decreasing processing procedures. The ultra-precision grinding method and etching with aqua regia method have been investigated for removing the Pt coating layer. Experimental results show that when the diamond cutting tool width is bigger than 500 nm, ultra-precision grinding method is appropriate for removing Pt coating layer on diamond tool. However, the ultra-precision grinding method is not recommended for removing the Pt coating layer when the cutting tool width is smaller than 500 nm, because the possibility that the diamond cutting tool is damaged by the grinding process will be increased. Despite the etching method requiring more procedures to remove the Pt coating layer after FIB milling, it is a feasible method for diamond tools with under 500 nm width.

## 1. Introduction

Freeform surfaces can be used in optical systems to achieve novel functions, improve performance, reduce size, and decrease the cost of various products. Optical freeform surfaces find applications in fields such as of optics, medicine, fiber communication, life science, and aerospace [1,2,3]. For instance, aspheric and Fresnel lenses can effectively improve image quality and chromatic aberrations and reduce the size of optical devices [4]. Lens arrays are effective for light integration and image improvement [5]. F-theta lenses are widely used in scanning systems due to their precision location characteristics [6]. Freeform optics has become a key element of quantitative light technology, which is becoming increasingly important in various fields [7,8]. To make these optical systems, micro/nanoscale single-crystal diamond cutting tools are essential. Pulsed laser ablation (PLA) and electric discharge machining (EDM) are the most common non-contact methods for macro/micro manufacturing of diamond devices [9], but they are not appropriate for manufacturing nanoscale diamond tools. 

In recent years, focused ion beam milling has been applied in sample preparation for transmission electron microscopy (TEM). Due to diamond’s extreme properties, it is very difficult to prepare a TEM sample from diamond using conventional preparation methods, including mechanical thinning, ion milling, and chemical etching [10,11]. FIB can be used to prepare a cross-sectional TEM sample of diamond, and it takes only a few hours. FIB is clearly a very useful technique for diamond processing at the micro and nanoscale levels which needs high accuracy. By using FIB milling, we obtained appropriate shaped micro/nano single-crystal diamond cutting tools. During the FIB milling, because of the Gaussian beam characteristics, the diamond could be damaged. For protecting the diamond during the cutting tool FIB milling process, a platinum (Pt) coating is essential as protection layer for milling accuracy and cutting tool edge sharpness. In this study, the appropriate Pt coating depth as FIB milling protection layer has been confirmed along with methods for removing Pt coating layer after diamond FIB milling.

## 2. Focused Ion Beam (FIB)

The basic operational principle of FIB devices is sputtering atoms from the target material by bombarding it with accelerated heavy ions. The efficiency of the sputtering process is mainly determined by the ion source, which must meet various requirements: the momentum transfer at a given acceleration voltage, usually *V* = 30 KeV, should be maximized by using heavy ions, and the source material should have a low melting point and a low vapor pressure. Both of these requirements are met by gallium.

Ga has a melting point of 29.8 °C. A Ga liquid metal ion source (LMIS) is composed of a small Ga reservoir connected to a tungsten needle. The solid Ga is heated to its melting point, and the liquid Ga flows to the tip of the tungsten needle by surface tension and wets it. A strong electric field *E* = 108 V/cm applied to the end of the tungsten tip causes the liquid Ga to form a point source with of about 2–5 nm in diameter and extracts ions from that the narrow tip. The Ga ions are accelerated in an electrical field *V* up to 30 KeV. A continuous flow of liquid Ga to the tip replaces the extracted Ga^+^ ions, resulting in a constant ion current, which is a basic requirement for an automated sputtering process [12].

## 3. Experimental Setup

**Figure 1:**
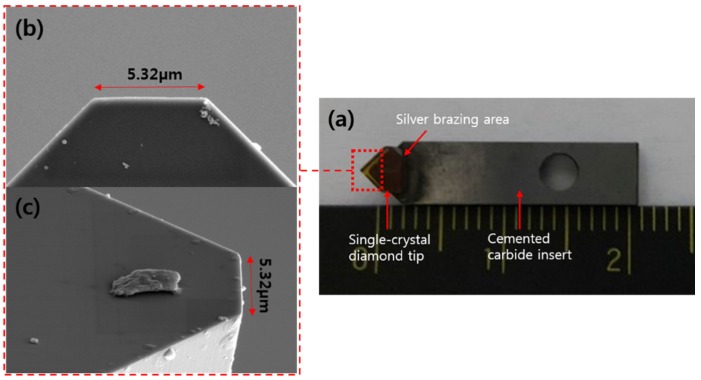
(**a**) Photograph of single-crystal diamond sample prepared with ultra-precision grinding; (**b**) SEM image of prepared diamond sample top view; (**c**) SEM image of prepared diamond sample side view.

An ultra-precision grinding machine and FIB were used in manufacturing micro/nanoscale single-crystal diamond tools. The PG3B ultra-precision planetary grinder (COBRON Engineering, Romford, UK) was used for preparing the diamond tool and removing Pt protection coating layer. The PG3B can make low-waviness tools down to 100 nm or better. Figure 1a shows the single-crystal diamond tip silver brazed on a cemented carbide insert. Figure 1b,c shows SEM images of the top and side view of single-crystal diamond prepared by ultra-precision grinding before FIB milling. The edge width of ultra-precision grinding prepared diamond tip is about 5.3 μm.

**Figure 2:**
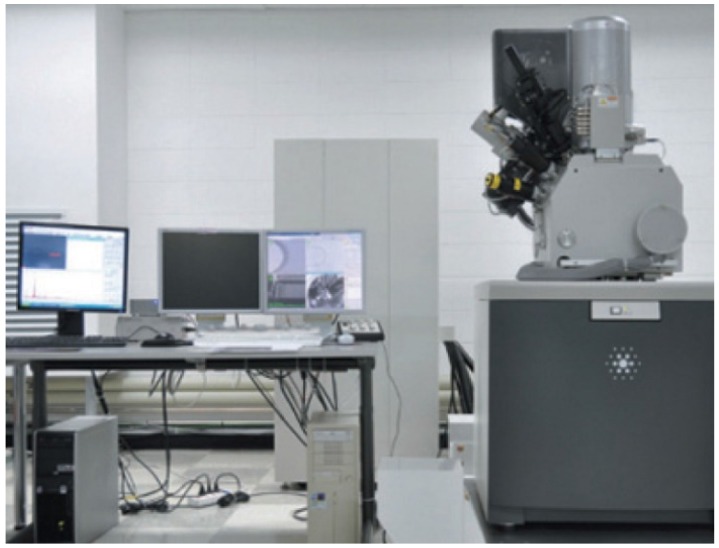
Photograph of focused ion beam system (FEI, NOVA600 Nanolab).

Experiments to develop micro/nanoscale single-crystal diamond tools were carried out using FIB (FEI COMPANY, system Nova 600 Nanolab, Hillsboro, OR, USA), as shown in Figure 2. The technique combines ultra-high resolution field emission scanning electron microscopy (SEM) and precise FIB milling, and it can be used in nanoscale prototyping, machining, characterization, and analysis of structures smaller than 100 nm. 

The FIB system uses a focused gallium ion beam working under an accelerating voltage ranging from 2 to 30 kV and probe current ranging from 1.5 pA to 60 nA. The resolution of the FE-SEM image is 1.1 nm. The tilting angle of the dual-beam system’s translation stage is −15 to 60°, and the rotational axis is employed to control the machining tool rotation continuously. Different tool faces can be milled by accurately adjusting their positions relative to the FIB by rotation and sample tilt control. In this experiment, the applied current ranges from 50 pA to 7 nA and the tilting angle is 52 ± 1° to 52 ± 6°.

## 4. Results and Discussion

### 4.1. Platinum Coating Depth

**Figure 3:**
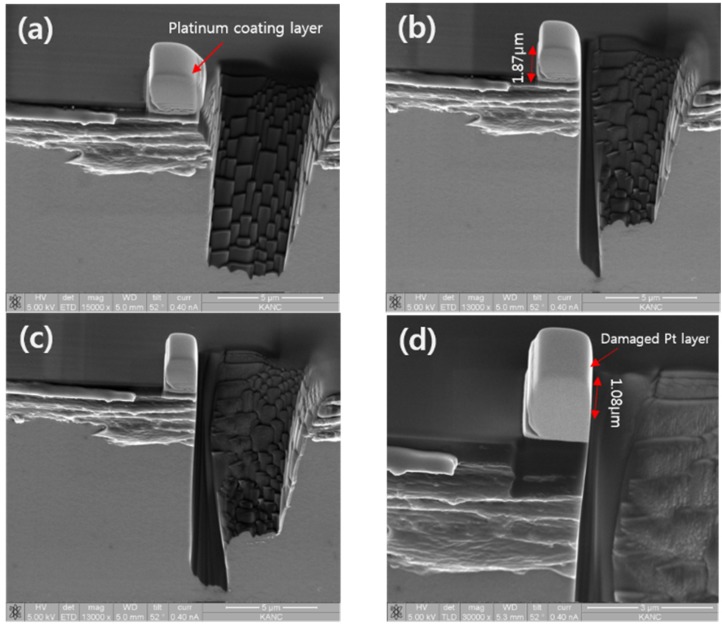
SEM images of (**a**) Rectangle type focused ion beam (FIB) machined diamond (tilt angle: 52 ± 4°, beam current: 7 nA, voltage: 30 kV); (**b**) Cleaning type FIB machined diamond (tilt angle: 52 ± 1.5°, beam current: 1 nA, voltage: 30 kV); (**c**) Cleaning type FIB machined diamond (tilt angle: 52 ± 1.5°, beam current: 100 pA, voltage: 30 kV); (**d**) Cleaning type FIB machined diamond (tilt angle: 52 ± 1.5°, beam current: 50 pA, voltage: 30 kV).

To successfully apply the single-crystal diamond protection layer, platinum was used. These coatings were obtained by FIB assisted deposition process. The coating depth of the platinum was 1.87 μm. Figure 3 shows SEM images of the platinum coating on a diamond tool and tests of the FIB-machined diamond. To make sure the platinum coating depth is appropriate, four FIB milling process were carried out. Rectangle type FIB milling with 7 nA beam current and 52 ± 4° tilt angle was applied as the first step, as shown in Figure 3a. For controlling the machining accuracy of the FIB milling, cleaning type FIB milling was applied and the beam current was reduced to 1 nA, 100 pA and 50 pA, as shown in Figure 3b–d. The tilt angle was 52 ± 1.5°. As shown, the platinum coating layer completely protected the diamond tool from FIB Gaussian characteristics. The damaged layer of the platinum coating is about 1.08 μm, as shown in Figure 3d. 

Moreover, the diamond retains a sharp cutting edge. Also, by controlling the tilt angle, the appropriate cutting tool clearance was obtained. In consideration of the processing time and cost, the platinum coating layer depth should be greater than 1.2 μm to protect the diamond. The experimental results revealed that the fabrication of micro/nanoscale diamond tools with Pt coating is an appropriate method for diamond FIB milling.

**Figure 4:**
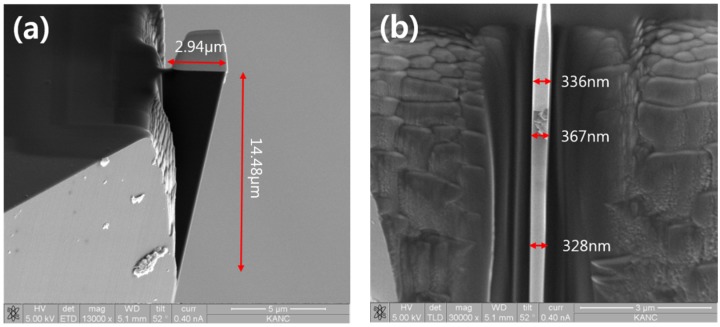
SEM images of 370 nm cutting edge width tool (FIB machined 2.3 μm Pt coating). (**a**) side view of sample (**b**) front view of sample.

Figure 4 shows the SEM images of the tool with cutting edge width and clearance under 370 nm fabricated by Pt coating and FIB milling. The Pt coating depth is 2.3 μm, and it protects the diamond cutting tool effectively. Beam currents of 3–7 nA and 52° were used with rectangle scan type FIB for initial diamond machining. The beam current was gradually reduced from 1 nA to 50 pA, tilting angle from 52 ± 6° to 52 ± 1° with cleaning scan type FIB to obtain a cutting edge width of approximately 370 nm. Obtaining such a cutting edge could require complicated procedures to obtain precise diamond tool geometry. Also, paying careful attention is required in order to obtain accurate cutting edge width. The cutting edge width and width/height ratio of about 1:5 satisfy the cutting tool requirements.

### 4.2. Removing Pt Coating Layer Using Ultra-Precision Grinding

**Figure 5:**
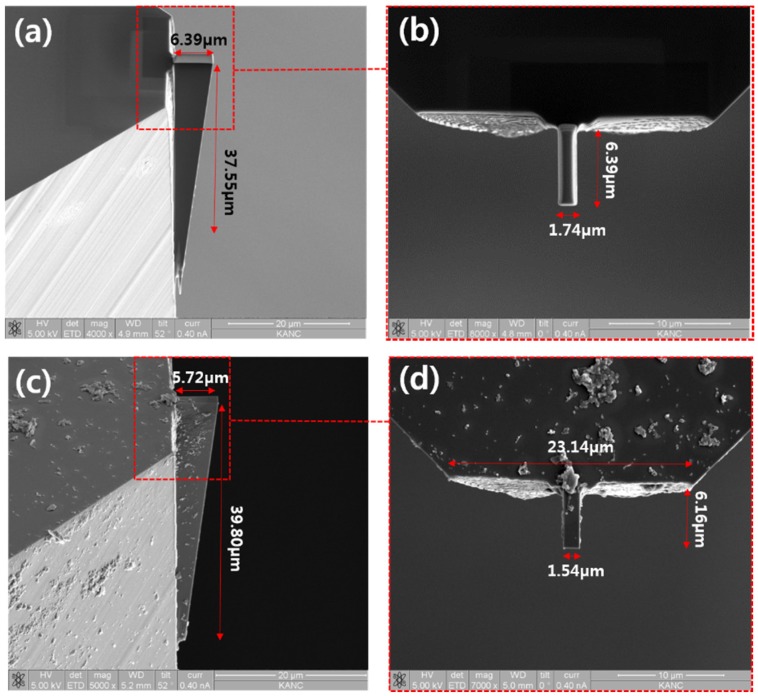
SEM images of 1.5 μm level FIB machined diamond cutting tool (**a**,**b**) before removing Pt coating layer; (**c**,**d**) after removing Pt coating layer using ultra-precision grinding.

An ultra-precision grinding machine, PG3B ultra-precision planetary grinder (COBRON Engineering) was used for preparing single-crystal diamond before FIB milling, and also used for removing Pt coating after FIB milling. Figure 5a,b shows the images of 1.5 μm level FIB machined diamond cutting tool before removing Pt coating layer using ultra-precision grinding and Figure 5c,d shows 1.5 μm level FIB machined diamond cutting tool after removing the Pt coating layer using ultra-precision grinding. In order to protect the diamond cutting tool, a Pt coating method was adopted. For manufacturing practicable cutting tool shape, rectangle type FIB milling was used as initial machining with an appropriate beam current and tilting angle. The appropriate beam current and tilting angle cleaning type FIB milling was used as precision and finish machining. After ultra-precision grinding, the cutting edge width of the diamond cutting tool is about 1.5 μm, and the height is about 40 μm.

**Figure 6:**
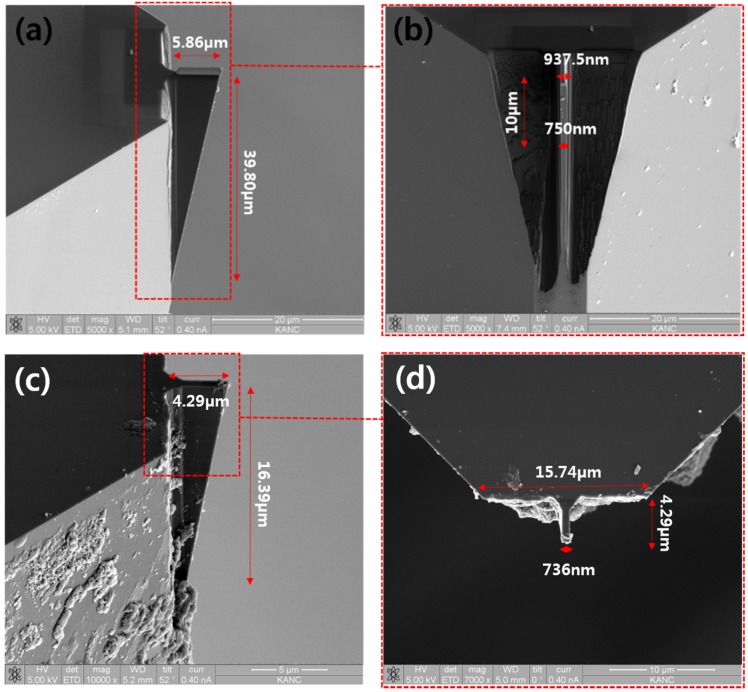
SEM images of 700nm level FIB diamond cutting tool (**a**,**b**) before removing Pt coating layer with ultra-precision grinding; (**c**,**d**) after removing Pt coating layer with ultra-precision grinding.

Cutting edge widths with a 730-nm diamond cutting tool are also obtained by removing the Pt coating layer with an ultra-precision grinding method, as shown in Figure 6. However, the grinding process could give damage to diamond cutting tool along with decrease of cutting edge width. When removing the Pt coating layer using the grinding method, there is no need to separate diamond tip from the insert and no need to increase unnecessary procedures which could lead to increasing process costs. Moreover, because of the grinding characteristics, the cutting edge sharpness of the diamond cutting tool will be improved. 

Experimental results show that ultra-precision grinding method is efficient Pt coating removing method. It can remove Pt coating stably when the diamond cutting width is greater than 700 nm. However, if the cutting tool width were smaller, the possibility of the diamond cutting tool being damaged by the grinding process will be increased. The ultra-precision grinding method is not appropriate for removing the Pt coating layer when the cutting tool width is smaller than 500 nm.

### 4.3. Removing Pt Coating Layer Using Etching with Aqua Regia 

**Figure 7:**
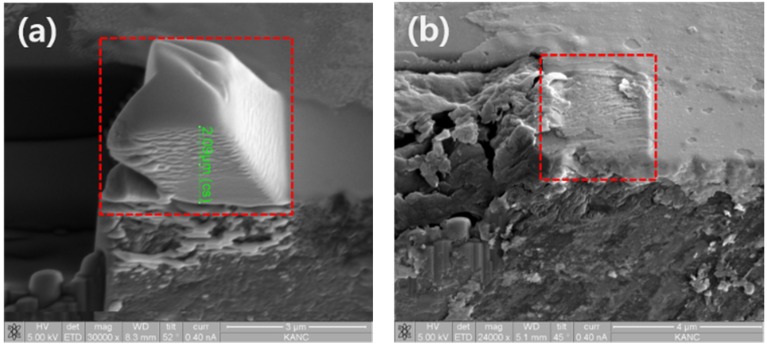
SEM images of (**a**) 2.09 μm Pt coating; (**b**) after etching with aqua regia for 8 h.

Aqua regia is a highly corrosive mixture of acids, a fuming yellow or red solution. The mixture is formed by freshly mixing concentrated nitric acid and hydrochloric acid, optimally in a volume ratio of 1:3. It can dissolve the noble metals gold and platinum. However, titanium, iridium, ruthenium, rhenium, tantalum, niobium, hafnium, osmium, rhodium and tungsten are capable of withstanding its corrosive properties. Figure 7a shows 2.09 μm Pt coating on single-crystal diamond for etching test with aqua regia acid. After etching with aqua regia for 8 h, the Pt protection layer has been almost removed, as shown in Figure 7b. Despite a relatively long etching time when compared with conventional machining method, it shows the possibility of removing the Pt layer with the etching method. 

**Figure 8:**
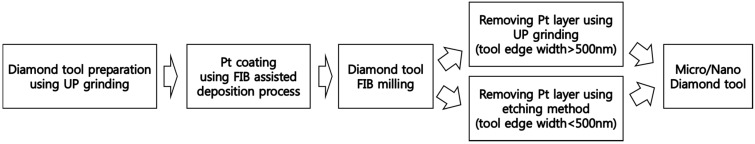
Block diagram of micro/nanoscale diamond cutting tool FIB milling process.

However, there are some problems to be fixed. First, the single-crystal diamond has been silver brazed on the insert. After FIB milling, to remove the Pt coating layer, the insert with the diamond is placed into the aqua regia acid. Not only the insert, but also the silver will be dissolved. Second, the diamond tip could be separated from the insert after FIB milling. However, the procedure could be very complex, and the accuracy of silver brazing diamond tip after etching is difficult to maintain. Third, due to the low dissolution rate of the Pt coating layer, more time would be needed compared with conventional machining method. Fourth, if there is a change in the material of the insert and diamond brazing method, it will be possible to etch the Pt coating without separating the insert and diamond. If the problems of removing the Pt coating layer with the etching method are solved, we can manufacture micro/nanoscale single-crystal diamond cutting tool as in the process shown in Figure 8.

## 5. Conclusions 

In this study, the depth of Pt coating layer which could decrease process-induced damage during FIB fabrication is investigated. Rectangle type FIB milling was used as initial machining for reducing diamond cutting tool fabrication time and cleaning type FIB milling was used as finish machining to obtain cutting tool sharpness and shape. To protect the diamond cutting tool from beam damage, the optimum Pt coating depth is at least 1.2 μm, and it is important to maintain the diamond cutting tool edge sharpness, which could decrease FIB milling procedures. With this method, a diamond cutting tool with a 370 nm edge width has been obtained. 

The ultra-precision grinding method is an appropriate method for removing Pt coating layer when the cutting edge width is bigger than 500 nm. A single-crystal diamond cutting tool edge with widths of 1.5 μm and 700 nm has been obtained. It has no need to separate diamond tip from insert which could decrease process costs. Moreover, due to the grinding process characteristics, the manufactured single-crystal diamond cutting tool edge sharpness will also be improved. However, along with the decrease of cutting edge width, the possibility of diamond cutting tool damaged by grinding process is increased. When removing the Pt coating layer under 500 nm cutting edge width the diamond tool, the grinding method is not suitable.

The etching with aqua regia method has been investigated for removing the Pt coating layer. Etching with aqua regia method shows the possibility of removing the Pt coating layer after FIB milling. However, the insert and silver would be dissolved when removing the Pt coating protection layer. The Pt removing time will be longer than conventional machining method, due to the low dissolution rate. By changing the material of insert which cannot dissolve by aqua regia (e.g., titanium, tungsten) and brazing method, etching could be a suitable method for removing a Pt coating protection layer under 500 nm cutting edge width a diamond tool.
